# Prescription of HIV Post-Exposure Prophylaxis in emergency care units and return for follow-up appointments in specialized services in Salvador, Brazil, 2018: a cross-sectional study

**DOI:** 10.1590/S2237-96222024V33E2023642.en

**Published:** 2024-07-15

**Authors:** Flávia Carneiro da Silva, Laio Magno, Carlos Antônio de Souza Teles Santos

**Affiliations:** 1 Prefeitura Municipal de Salvador, Secretaria Municipal de Saúde, Salvador, BA, Brazil; 2 Universidade do Estado da Bahia, Departamento de Ciências da Vida, Salvador, BA, Brazil; 3 Fundação Oswaldo Cruz, Centro de Integração de Dados e Conhecimentos para Saúde, Salvador, BA, Brazil

**Keywords:** HIV, Post-Exposure Prophylaxis, Disease Prevention, Access to Essential Medicines and Technologies, Continuity of Patient Care, Cross-Sectional Studies, VIH, La Profilaxis Post Exposición, Prevención de Enfermedades, Acceso a Medicamentos y Tecnologías Esenciales, Continuidad de la Atención al Paciente, Estudios Transversales

## Abstract

**Objective:**

To analyze HIV Post-Exposure Prophylaxis (PEP) prescription and return for follow-up appointments.

**Methods:**

This was a descriptive cross-sectional study using data on people who sought PEP in emergency care units (UPAs) and specialized medical services in Salvador, BA, Brazil, between January-December/2018.

**Results:**

Of the 1,525 people who sought PEP at UPAs, 1,273 (83.5%) met PEP eligibility criteria, while 252 (16.5%) did not; of the eligible group, 1,166 (91.6%) had antiretrovirals prescribed, while 107 (8.4%) eligible people did not; of the total number of people with PEP prescriptions, only 226 (19.4%) returned for the first follow-up appointment, 115 (9.9%) for the second, and 33 (2.8%) for the third in order to complete the protocol.

**Conclusion:**

We found a significant proportion of eligible users who did not have PEP prescribed at UPAs and a significant loss of return for specialized service follow-up appointments.

## Study contributions

**Main results:**

Of those eligible for post-exposure prophylaxis (PEP), 8.4% did not receive it in emergency care units (UPAs); of those who did receive it, only 19.4% returned for the 1^st^ appointment, 9.9% for the 2^nd^ appointment and 2.8% for the 3^rd^ appointment in order to complete the protocol.

**Implications for services:**

Training and discussion of the implementation of initial prescription of PEP at UPAs and its subsequent monitoring in centers specialized in the human immunodeficiency virus (HIV) must be promoted; percentage adherence to follow-up was low.

**Perspectives:**

Expansion of continuing education actions for UPA professionals to ensure timely PEP prescription, in addition to the creation of strategies to link PEP users to follow-up appointments in health services.

## Resumen

**Objetivo:**

Analizar la prescripción de Profilaxis Post Exposición (PPE) al Virus de la Inmunodeficiencia Humana (VIH) y la concurrencia a las consultas de control y seguimiento.

**Método:**

Estudio de datos descriptivo transversal de personas que solicitaron la PPE en Unidades de Atención de Emergencia (UPA) y servicios médicos especializados en Salvador, BA, Brasil, durante los meses de enero a diciembre de 2018.

**Resultados:**

De las 1.525 personas que consultaron por PEP en las unidades hospitalarias de emergencia, 1.273 (83,5%) cumplieron criterios de elegibilidad para profilaxis y 252 (16,5%) no los cumplieron; entre los elegibles, a 1.166 (91,6%) personas se les recetaron antirretrovirales (ARVs), pero 107 (8,4%) no los recibieron. Del total de personas que recibieron prescripción de PPE, solamente 226 (19,4%) regresaron a la primera consulta; 115 (9,9%) a la segunda consulta y 33 (2,8%) a la tercera consulta para completar el protocolo.

**Conclusión:**

Hubo una proporción significativa de usuarios elegibles que no recibieron prescripción de PEP en las Unidades de Atención de Emergencia y una pérdida significativa en las consultas de control al servicio especializado.

## INTRODUCTION

Infection with the human immunodeficiency virus (HIV) is a national and global public health problem. Efforts to contain acquired immunodeficiency syndrome (AIDS), a disease caused by this pathogen, have resulted in the creation of increasingly effective treatments. Expansion of the supply of antiretroviral therapy, in turn, has changed the prognosis people living wth HIV, whereby it is now considered to be a treatable chronic infection.^1^ ^),(^ ^2^

The emergence of prevention methods based on the use of antiretrovirals (ARVs), linked to counseling, testing and condom distribution, has brought encouragement and belief in the end of the epidemic.^2^ ^)-(^ ^4^ The combined use of HIV prevention strategies can reduce the risk of transmissibility of the virus. These treatments have been shown to be effective in viral suppression and, in reducing HIV incidence.^5^ ^),(^ ^6^

A set of combination prevention strategies, which incorporate behavioral, structural and biomedical approaches, have been adopted with the aim of reducing HIV incidence in Brazil.^7^ The biomedical approach relies on the prescription of ARVs to prevent new infections, through two types of prophylaxis: (i) HIV pre-exposure prophylaxis and HIV post-exposure prophylaxis (PEP).^8^ ARVs demonstrate high efficacy in preventing HIV infection after risk exposure.^2^ ^),(^ ^9^ ^)-(^ ^11^ PEP, for example, constitutes the last HIV prevention measure and can be applied within a period from the moment the person is exposed to the virus until it reaches the regional lymph nodes, an interval of time that can last up to 72 hours. Experimental models suggest that the sooner ARVs are administered, the greater the effectiveness of PEP in preventing HIV transmission.^12^ ^),(^ ^13^

In Brazil, PEP has been recommended by the Ministry of Health since the 1990s.^7^ Initially indicated in cases of occupational accidents, with exposure to biological material, this prophylaxis began to be used to prevent vertical transmission and, later, to treat cases of sexual violence.^14^ ^)-(^ ^17^ With effect from 2010, the “Clinical Protocol and Treatment Guidelines for Post-Exposure Prophylaxis for Risk of HIV Infection” (“*Protocolo Clínico e Diretrizes Terapêuticas da Profilaxia Pós-Exposição ao Risco de Infecção pelo HIV*”) regulated provision of PEP for use in consented sexual intercourse.^18^ In 2015, both indication of PEP and the medication regimen were simplified, becoming based on assessment of risk of exposure regardless of the type of sexual partners or sexual practice;^19^ in 2017, the ARVs dolutegravir was included in PEP;^7^ in 2018, a strategy was launched to facilitate prescription by non-specialist medical professionals, at the initial stage of assessment, and to include an expanded approach in relation to other sexually transmitted infections (STIs) and viral hepatitis;^8^ and, in 2021, the Ministry of Health updated the guidelines for expanding the provision of prophylaxis in urgent and emergency services, primary health centers, clinics and hospitals in the public and private network, aided by a Federal Council of Nursing (*Conselho Federal de Enfermagem*) official document in support of PEP being prescribed by nurses.^20^

Implementing biomedical HIV prevention interventions faces structural and individual challenges, ranging from insufficient training of health professionals to lack of knowledge of PEP itself, low perception of risk of infection, insufficient adherence to prophylaxis and to follow-up appointments.^2^ ^),(^ ^21^

The objective of this study was to analyze prescription PEP and return for follow-up appointments in the city of Salvador, Bahia, Brazil.

## METHODS

This is a descriptive cross-sectional study, carried out in the city of Salvador, capital of the state of Bahia. Salvador is the largest city in the Northeast macro-region of Brazil, with almost three million inhabitants, mostly self-reported as Black and mixed race. Furthermore, the city is the main provider of Brazilian National Unified Health System (*Sistema Único de Saúde* - SUS) services in the state, ranging from primary to specialized and high complexity care.

In Bahia, the AIDS detection rate remained stable between 2009 and 2019, varying from 12.6 to 12.9 per 100,000 inhabitants in that period. For Salvador, official data indicate a falling trend, from 27.9 AIDS cases per 100,000 inhab., in 2009, to 24.4 cases per 100,000 inhab. in 2019.^22^

The study population consisted of all people who sought PEP in Salvador, from January 1st to December 31^st^, 2018. The criteria for indicating PEP were: seeking PEP within 72 hours after exposure; having been exposed to HIV and testing non-reactive; and the source person testing reactive for HIV or having unknown HIV status. It is important to highlight that HIV testing in emergency care units (*unidades de pronto atendimento* - UPAs) was carried out using a third-generation rapid test, for the qualitative detection of HIV antibodies. Advantages of using rapid testing include it being easy to perform, no need for a complex laboratory structure, reading of results with the naked eye and completion within 30 minutes. The seroconversion window period for third-generation assays is approximately 22 to 25 days.^23^

According to the PEP care protocol in the city of Salvador, in 2018, when an individual was exposed to HIV, they went to one of the city’s UPAs for initial assessment of the time and type of exposure; following this rapid tests for HIV, syphilis, hepatitis B and C were performed, which would also be offered to the source person if they were present. Once this initial assessment was completed, the procedures regarding PEP being prescribed for HIV and/or other STIs were defined. Following this, ARVs were dispensed for use during 28 days, and the individual was given guidance and referred for follow-up at the Specialized Medical Care Service (*Serviço Médico da Atenção Especializada* - SEMAE), located in the Liberdade Health District.

For the purposes of follow-up and treatment continuity, individuals who received PEP as prescribed were recommended to attend three follow-up appointments at the SEMAE, scheduled for two weeks, 30 days and 90 days after the date of exposure. At the first appointment, the individual was assessed regarding ARVs toxicity, with the aim of identifying possible adverse effects; and guidance was provided to reinforce their adherence to prophylaxis. At the 30 and 90 day post-exposure follow-up appointments, new HIV tests were performed and guidance on infection prevention measures was reinforced, such as the use of condoms in all sexual relations and not sharing syringes and needles (in cases of use of injectable drugs), in addition to contraindication of donating blood, organs, tissues or sperm, and reaffirming the importance of avoiding pregnancy during treatment.^8^ Follow-up appointments at the SEMAE were carried out by a multidisciplinary team, consisting of a social worker, a nurse, a pharmacist, an infectious disease specialist doctor, a psychologist and a nursing technician.

The study was conducted based on secondary data. Access to and construction of the anonymized database took place in two stages.

In the first stage, between December 2, 2019 and January 31, 2020, a database of service users who sought PEP was built in the five UPAs that had a PEP protocol implemented in the city of Salvador, in 2018 (i.e., Barris, Marback, Hélio Machado, Albergaria and Valéria). Service user information was organized on PEP Monitoring Spreadsheets and was provided by the STI/Aids and Viral Hepatitis Monitoring Sector of the Salvador City Health Department.

The PEP Monitoring Spreadsheets were filled out by nurses from the UPA epidemiology centers, according to the information contained in the care records. Standing out among the information recorded on the PEP Monitoring Spreadsheet are: the patient’s name, age, sex, date of initial PEP care/evaluation, probable date of exposure to HIV, time elapsed since exposure (period, in hours, from probable exposure to seeking care at the UPA), type of exposure, rapid tests carried out during care and their respective results, and PEP prescribed. No record was made of service user gender identity. The standardized protocol for filling out the PEP Monitoring Spreadsheets was prepared by the Epidemiological Surveillance service of the Salvador City Health Department. The database for this stage was created by compiling all the information cataloged at the five UPAs, taken from the PEP Monitoring Spreadsheets. A total of 60 spreadsheets were compiled, covering the 12 months of 2018 (January 1 to December 31, 2018), filled out at the five emergency care units mentioned previously. The database was anonymized using sequential identifier codes. We used the SPSSwin 22.0 and Excel programs for the database pre-processing phase.

The second stage of building the database consisted of data collection from SEMAE records, i.e. the service where the follow-up appointments took place. We collected data from the records of the first, second and third PEP follow-up appointments. In this stage, service users were identified by name to locate their medical records, and then coded with the identifier code assigned in the first stage. A collection instrument was used, aiming to standardize data recording. We collected sociodemographic data, type of HIV exposure, name of the UPA where the first service was provided, as well as data from the first, second and third follow-up appointments. This stage of data collection took place between June 1, 2020 and June 23, 2020.

The variables analyzed were:

a) PEP prescribed (yes; no);

b) attending all PEP follow-up appointments at the SEMAE (yes; no);

c) sex (male; female);

d) age (in years: under 18; 18-23; 24-33; 34 or over);

e) type of exposure (consented sexual intercourse; accidental exposure to biological material; sexual violence);

f) time elapsed since exposure (up to 72 hours; more than 72 hours);

g) HIV screening test result (non-reactive; reactive);

h) syphilis screening test result (non-reactive; reactive);

i) hepatitis B screening test result (non-reactive; reactive);

j) hepatitis C screening test result (non-reactive; reactive);

k) PEP eligibility (eligible; not eligible);

l) reasons for PEP not being prescribed for eligible people (refusal; left against medical advice; medical professional did not prescribe it; lack of ARVs at the UPA; no information);

m) PEP adverse effects (nausea; diarrhea; abdominal pain; headache; vomiting; jaundice; dry mouth); and

n) PEP maintenance (yes; suspension after source person testing non-reactive for HIV).

We performed descriptive analysis of the variables of interest, obtaining absolute and percentage frequencies. We used Stata version 12.0 (StataCorp LP, College Station, USA) for the statistical analyses.

Access to the PEP Monitoring Spreadsheets and SEMAE records was granted by Term of Consent No. 79/2019, provided by the Health Personnel Management Sector of the Salvador City Health Department, authorizing the use of the information for this research. The research project was approved by the Research Ethics Committee of the Instituto de Saúde Coletiva of the Universidade Federal da Bahia: Certificate of Submission for Ethical Appraisal (Certificado de Apresentação para Apreciação Ética - CAAE) No. 25525719.6.0000.5030; Opinion No. 3,746,970 aproved on december 05, 2019. Confidentiality of primary data was guaranteed during collection and the resulting anonymous database was analyzed on a password protected restricted use private computer not connected to the internet in order to protect the information.

## RESULTS

Of the 1,550 PEP consultations registered in 2018, 25 were excluded because they referred to records of source persons of individuals who suffered an accident with biological material and, therefore, did not require PEP. Of the 1,525 people who sought PEP at UPAs in the city of Salvador, 1,056 (69.2%) were male and 469 (30.8%) were female. The median age of these people was 28 years old (interquartile range: 22 to 35), with a predominance of care provided to the 24-33 year age group (39.4%). Among childrens and adolescents < 18 years old, there was a total of 81 consultations (5.3%) (Table 1), of which 37 (45.7%) were due to sexual violence, 24 (29.6%) due to consented sexual intercourse and 20 (24.7%) due to needlestick accidents.

**Table 1:** Demographic characteristics and characteristics of exposure to human immunodeficiency virus (HIV) infection and procedures performed during assessment of post-exposure prophylaxis following risk of infection, Salvador, Bahia, Brazil, 2018

Characteristics	N	%
Sex		
Male	1,056	69.2
Female	469	30.8
Age (years)		
< 18	81	5.3
18-23	398	26.1
24-33	601	39.4
≥ 34	440	28.9
No information	5	0.3
Type of HIV exposure		
Consented sexual intercourse	1,164	76.3
Accidental exposure to biological material	270	17.7
Sexual violence	86	5.6
No information	5	0.3
Time since (hours)		
≤ 72	1,381	90.6
> 72	93	6.1
No information	51	3.3
HIV		
Non-reactive	1,403	92.0
Reactive	25	1.6
Not performed	97	6.4
Syphilis		
Non-reactive	1,265	83.0
Reactive	156	10.2
Not performed	104	6.8
Hepatitis B		
Non-reactive	1,407	92.3
Reactive	2	0.1
Not performed	116	7.6
Hepatitis C		
Non-reactive	1,408	92.3
Reactive	2	0.1
Not performed	115	7.5
HIV post-exposure prophylaxis (PEP) Indication		
Yes	1,273	83.5
No	252	16.5
HIV post-exposure prophylaxis (PEP) Prescription		
Yes	1,166	91.6
No	107	8.4
Reasons for HIV post-exposure prophylaxis (PEP) not being prescribed for people with indication		
Refusal	42	39.2
Left against medical advice	37	34.6
Medical professional did not prescribe it	22	20.6
Lack of ARVs at the emergency care unit	3	2.8
No information	3	2.8

Regarding the type of exposure that led people to seek PEP, 1,164 (76.3%) reported unprotected sexual contact, 270 (17.7%) accidental exposure to biological material and 86 (5.6%), sexual violence. Among men, forms of exposure that led them to seek PEP included unprotected sexual contact (938 = 88.8%), accident with exposure to biological material (101 = 9.6%), sexual violence (12 = 1.1% ) and the reason for seeking PEP not being recorded (5 = 0.5%); while among women, forms of exposure and reasons for seeking PEP were unprotected sexual contact (226 = 48.2%), accident with exposure to biological material (169 = 36%) and sexual violence (74 = 15.8%) (Table 1).

The time elapsed from the moment of exposure until arrival at the UPA was less than or equal to 72 hours for 1,381 individuals (90.6%); 93 (6.1%) sought PEP after this period. Taking all of them, 1,403 (92.0%) had a non-reactive HIV test during the first visit; the test result was reactive in only 25 of them (1.6%), who were then referred for treatment at the SEMAE. Finally, 156 (10.2%) had a reactive syphilis test, 2 (0.1%) for hepatitis B and 2 (0.1%) for hepatitis C (Table 1).

Of the total number of people who sought PEP, 1,273 (83.5%) met the eligibility criteria for starting PEP immediately, while 252 (16.5%) did not meet the criteria. Among the reasons for ineligibility, the majority sought the service more than 72 hours after exposure (93 = 36.9%). It should be noted that 25 received a reactive HIV test result at the first consultation (9.9%), for 84 of them, the source person tested non-reactive for HIV (33.3%) and 50 had no record of the reason for ineligibility in their medical records. (19.9%).

Considering the people for whom PEP was indicated, 1,166 (91.6%) had ARVs prescribed and were advised to adhere to the follow-up provided for in the protocol. However, 107 (8.4%) of them did not receive a prescription, despite meeting the indicated PEP criteria, and the reasons for not prescribing PEP were: 42 (39.2%) refused prophylaxis; 37 (34.6%) left the UPA against medical advice; 22 (20.6%) did not have ARVs prescribed by the medical professional; and 3 (2.8%) did not receive a prescription due to lack of medication at the UPA (Table 1).

**Figure 1:**
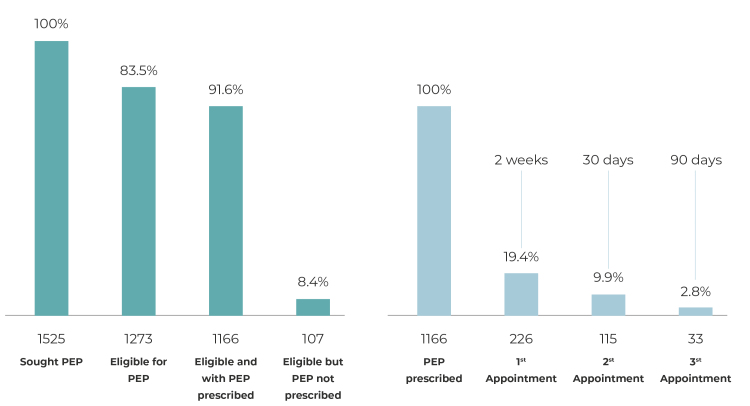
Prescription of post-exposure prophylaxis following risk of human immunodeficiency virus (HIV) infection and return for follow-up appointments, Salvador, Bahia, Brazil, 2018

Of the people who did receive PEP prescribed for them (1,166), 226 (19.4%) attended the first follow-up appointment, 115 (9.9%) the second appointment and 33 (2.8%) the third appointment in order to complete the protocol (Figure 1). All users who attended follow-up appointments were tested for HIV on those occasions, with non-reactive results. The majority who attended the first follow-up appointment at the SEMAE were male (64.6%), aged 34 or over (37.2%), and had been exposed to risk of infection through consented sexual intercourse (70. 4%). In the first follow-up appointment, the most frequent adverse reactions to ARVs were nausea (32.7%), diarrhea (19.9%), abdominal pain (7.1%), headache (6.2%), vomiting (3.5 %), jaundice (3.5%) and dry mouth (2.2%). The majority adhered to the ARVs regimen (96.9%) and only 3.1% were advised to suspend PEP due to the source person’s HIV test having a non-reactive result (Table 2).

**Table 2:** Demographic characteristics and characteristics of exposure to human immunodeficiency virus (HIV) infection and characteristics related to first follow-up appointments at the Specialized Medical Care Service, and frequency of attending appointments, Salvador, Bahia, Brazil, 2018

Characteristics	N	%
Sex		
Male	146	64.6
Female	80	35.4
Age (in years)		
< 18 18-23	13 53	5.8 23.5
24-33	76	33.6
≥ 34	84	37.2
Exposure type		
Consented sexual intercourse	159	70.4
Accidental exposure to biological material	54	23.9
Sexual violence	13	5.8
Adverse effects		
Nausea	74	32.7
Diarrhea	45	19.9
Abdominal pain	16	7.1
Headache	14	6.2
Vomiting	8	3.5
Jaundice	8	3.5
Dry mouth	5	2.2
HIV post-exposure prophylaxis (PEP) maintained		
Yes	219	96.9
Suspended after source person had non-reactive HIV test	7	3.1
1^st^ follow-up appointment	226	19.4
2^nd^ follow-up appointment	115	9.9
3^rd^ follow-up appointment	33	2.8

a) PEP: HIV post-exposure prophylaxis (HIV = human immunodeficiency virus).

## DISCUSSION

PEP is a safe prevention strategy and plays an even broader role in HIV testing and diagnosis of other STIs. In this study, when analyzing prescription of PEP and return for follow-up appointments in the city of Salvador, in 2018, the results described that PEP was sought at in UPAs mainly by younger people (24 to 33 years old), males, and those exposed to infection in consented sexual intercourse. However, subsequent follow-up at the SEMAE recorded a profile of older people (34 years or older). Furthermore, the percentage of people who adhered to the full PEP protocol in force at the time, that is, attending the three follow-up appointments, reduced over time, from 19.4% to 2.8% of the total number of people who started prophylaxis.

The findings of this research corroborate those of other studies, by demonstrating that prevention methods based on ARVs are sought more by young and male people, with a similar average age.^10^ ^),(^ ^24^ A cohort study conducted at Tel Aviv, Israel, between January 2013 and June 2014, identified 75.9% of people who sought PEP as being adult men who have sex with men with an average age of 32.4 years.^24^ In our study in Salvador, sexual exposure was also the main reason for seeking PEP, corroborating another study, conducted in a community clinic in the city of Los Angeles, United States, between March 2010 and July 2014.^25^

Despite PEP being a consolidated prevention strategy, some health system users refused it or left the unit against medical advice, while in other cases, the medical professional did not prescribe ARVs for them. These findings point to failures in reception by health services; as was also found by another study, conducted with 4,188 female sex workers, recruited through respondent-driven sampling, in 12 Brazilian cities, in 2016. In that study, 1,199 women reported having suffered sexual violence and only 7.5% of them sought medical assistance and used PEP; and 19%, even though they sought medical assistance, did not use PEP.^26^

The creation of training programs for health professionals at UPAs and other services that offer PEP via the SUS is important, so that they consider this technology, both as a medical emergency and as a process, and recommend a welcoming attitude on the part of health professionals free from value judgments.^27^ ^),(^ ^28^

With regard to follow-up appointments, the findings of this research reveal a drop between those who had PEP prescribed and their attending follow-up appointments at the specialized reference service. Failure to have follow-up can imply difficulties for follow-up, and for adherence to PEP^2^ ^)-(^ ^4^ during the 28 day period, and ultimately, the success of the strategy.^8^ It is important to highlight that UPAs and SEMAE did not actively go in search of service users who failed to attend follow-up appointments, an action that could contribute to the effectiveness of PEP.

Although it was not possible to identify the reasons for loss to follow-up, there is a hypothesis that fragmentation of PEP care in the city of Salvador, that is, the initial service provided in one health care unit and follow-up in another - often separated by kilometers of distance - played an important role in preventing individuals from being linked to the specialized follow-up service. A study carried out in the city of Boston, United States, in 2014, found that people treated in an emergency unit who received 3 to 6 days of for PEP and subsequently referred for follow-up at a specialized clinic, had low adherence to this latter service.^29^ Several factors can explain this difficulty, such as, for example, the characteristics of the health services, the quality of care, the relationship between the health professional and the service user, in addition to the accessibility of the service.

This study has some limitations. By being based on secondary data, extracted from spreadsheets and medical records filled out by health service professionals subject to the demands of an emergency unit, the quality of the information relies, on the one hand, on the willingness and judgment of those professionals, when inquiring about and recording important information and, on the other hand, the willingness of individuals to report intimate information about themselves. Furthermore, the number of variables available was small, as the data came from service records and not from previously designed research to collect this data. Therefore, the database built for the study may not have included important variables for analysis, such as people’s gender identity and issues of accessibility to specialized services. However, the results found point to the need to increase the dissemination of information about PEP, as the municipality offers this treatment free of charge on the SUS, in emergency care units open 24 hours a day.

In conclusion, our study indicates the need to reevaluate the implementation process in the city of Salvador, through qualification of professionals in charge of PEP care, dissemination, of the places where people can access prophylaxis and team to follow up on those using PEP, via telephone calls and/or applications, in order to expand and qualify access to treatment technologies based on ARVs. These are, precisely, actions that can contribute to consolidating the principles and strengthening the SUS, addressing and overcoming social stigmas, reaffirming the political struggle for the right to health in Salvador and in Brazil.^30^
