# Supplementary material for: Prescription of HIV Post-Exposure Prophylaxis in emergency care units and return for follow-up appointments in specialized services in Salvador, Brazil, 2018: a cross-sectional study
Source: Epidemiol Serv Saude. 2024 Jul 15;33:e2023642. doi: 10.1590/S2237-96222024V33E2023642.en (PMC11251451; doi:10.1590/S2237-96222024V33E2023642.en)
Supplement: Translation

# Translation: Prescrição da profilaxia pós-exposição ao HIV em unidades de urgência e retorno às consultas de acompanhamento em serviços especializados, Salvador, 2018: um estudo transversal

**DOI:** 10.1590/S2237-96222024v33e2023642.pt

**Authors:** Flávia Carneiro da Silva, Laio Magno, Carlos Antônio de Souza Teles Santos

**Affiliations:** 1 Prefeitura Municipal de Salvador, Secretaria Municipal de Saúde, Salvador, BA, Brazil; 2 Universidade do Estado da Bahia, Departamento de Ciências da Vida, Salvador, BA, Brazil; 3 Fundação Oswaldo Cruz, Centro de Integração de Dados e Conhecimentos para Saúde, Salvador, BA, Brazil

**Keywords:** HIV, Profilaxia Pós-Exposição, Prevenção de Doenças, Acesso a Medicamentos Essenciais e a Tecnologias, Continuidade da Assistência ao Paciente, Estudos Transversais

## Abstract

**Objetivo:**

Analisar a prescrição da profilaxia pós-exposição (PEP) ao vírus da imunodeficiência humana (HIV) e o retorno às consultas de acompanhamento.

**Métodos:**

Estudo transversal descritivo, sobre dados de pessoas que buscaram a PEP em unidades de pronto atendimento (UPAs) e serviço médico de atenção especializada em Salvador, BA, Brasil, entre janeiro e dezembro/2018.

**Resultados:**

Das 1.525 pessoas que buscaram a PEP nas UPAs, 1.273 (83,5%) apresentaram critérios de elegibilidade à profilaxia e 252 (16,5%) não os apresentaram; entre os elegíveis, 1.166 (91,6%) pessoas tiveram prescrição dos antirretrovirais (ARVs), mas 107 (8,4%) não os receberam; do total de pessoas com prescrição de PEP, apenas 226 (19,4%) retornaram à primeira consulta, 115 (9,9%) à segunda consulta e 33 (2,8%) à terceira consulta para conclusão do protocolo.

**Conclusão:**

Observou-se proporção importante de usuários elegíveis que não receberam prescrição de PEP nas UPAs e perda significativa nas consultas de retorno ao serviço especializado.

## Contribuições do estudo

**Principais resultados:**

Dos elegíveis para profilaxia pós-exposição (PEP), 8,4% não a receberam nas unidades de pronto atendimento (UPAs); dos que a receberam, apenas 19,4% retornaram à 1^a^ consulta, 9,9% à 2^a^ consulta e 2,8% à 3^a^ consulta para conclusão do protocolo.

**Implicações para os serviços:**

Deve-se promover treinamento, discussão da implementação da prescrição inicial da PEP nas UPAs e seu acompanhamento posterior nos centros de especialidades no vírus da imunodeficiência humana (HIV); foi baixo o percentual de adesão ao acompanhamento.

**Perspectivas:**

Ampliação das ações de educação permanente dos profissionais das UPAs para a prescrição da PEP de maneira oportuna, além da criação de estratégias de vinculação dos usuários de PEP às consultas de acompanhamento nos serviços.

## INTRODUÇÃO

A infecção pelo vírus da imunodeficiência humana (HIV) é um problema de saúde pública no âmbito nacional e mundial. Os esforços para conter a síndrome da imunodeficiência adquirida (aids), doença causada por esse patógeno, resultaram na criação de tratamentos cada vez mais eficazes. A ampliação da oferta da terapia antirretroviral, por sua vez, modificou o prognóstico pessoas vivendo com HIV, passando-se a considerá-la uma infecção crônica tratável.^1^ ^),(^ ^2^

O surgimento de métodos de prevenção baseados no uso de antirretrovirais (ARVs), atrelado ao aconselhamento, testagem e distribuição de preservativos, trouxe ânimo e crença no fim da epidemia.^2^ ^)-(^ ^4^ O uso combinado de estratégias de prevenção do HIV pode reduzir o risco de transmissibilidade do vírus. Tais tratamentos se mostraram eficazes na supressão viral e redução da incidência do HIV.^5^ ^),(^ ^6^

Um conjunto de estratégias de prevenção combinada, que incorporam abordagens comportamentais, estruturais e biomédicas, vem sendo adotado com o objetivo de reduzir a incidência do HIV no Brasil.^7^ A abordagem biomédica conta com a prescrição de ARVs para evitar novas infecções, mediante dois tipos de profilaxia: a profilaxia pré-exposição e a profilaxia pós-exposição (PEP) ao HIV.^8^ Os ARVs apresentam alta eficácia na prevenção da infecção pelo HIV após uma exposição de risco.^2^ ^),(^ ^9^ ^)-(^ ^11^ A PEP, por exemplo, constitui a última medida de prevenção do HIV e pode ser aplicada dentro de um período de prevenção, a contar do momento em que a pessoa é exposta ao vírus até este alcançar os linfonodos regionais, intervalo de tempo que pode durar até 72 horas. Modelos experimentais sugerem que, quanto mais rápida for a implementação de ARVs, maior será a efetividade da PEP na prevenção da transmissão do HIV.^12^ ^),(^ ^13^

No Brasil, a PEP é preconizada pelo Ministério da Saúde desde a década de 1990.^7^ Inicialmente indicada em casos de acidente ocupacional, com exposição a material biológico, essa profilaxia passou a ser utilizada na prevenção da transmissão vertical e, posteriormente, no atendimento aos casos de violência sexual.^14^ ^)-(^ ^17^ A partir de 2010, o “Protocolo Clínico e Diretrizes Terapêuticas da Profilaxia Pós-Exposição ao Risco de Infecção pelo HIV” regulamentou a oferta da PEP em relações sexuais consentidas.^18^ Em 2015, houve uma simplificação da indicação da PEP, assim como do esquema medicamentoso, que passou a se basear na avaliação do risco da exposição independentemente do tipo de parceria ou da prática sexual;^19^ em 2017, a PEP incluiu o antirretroviral dolutegravir;^7^ em 2018, foi lançada uma estratégia para facilitar a prescrição por profissionais médicos não especialistas, no momento inicial da avaliação, e incluir uma abordagem ampliada em relação às demais infecções sexualmente transmissíveis (ISTs) e hepatites virais;^8^ e em 2021, o Ministério da Saúde atualizou as diretrizes de ampliação da oferta de profilaxia em serviços de urgência e emergência, unidades básicas de saúde (UBS), clínicas e hospitais da rede pública e privada, inclusive com a contribuição do Conselho Federal de Enfermagem em parecer que respaldou a prescrição da PEP por enfermeiros.^20^

A implementação de intervenções biomédicas de prevenção do HIV enfrenta desafios estruturais e individuais, do insuficiente treinamento dos profissionais de saúde ao desconhecimento da própria PEP, baixa percepção de risco de infecção, insuficiente adesão à profilaxia e às consultas de acompanhamento.^2^ ^),(^ ^21^

O presente estudo teve como objetivo analisar a prescrição da PEP e o retorno às consultas de acompanhamento no município de Salvador, Bahia, Brasil.

## MÉTODOS

Trata-se de estudo transversal descritivo, realizado no município de Salvador, capital do estado da Bahia. Salvador é a maior cidade da macrorregião Nordeste do Brasil, com quase três milhões de habitantes majoritariamente autodeclarados pretos e pardos. Além disso, a cidade representa a principal oferta de serviços do Sistema Único de Saúde (SUS) no estado, da atenção primária à especializada e de alta complexidade.

Na Bahia, a taxa de detecção de aids manteve-se estável entre 2009 e 2019, variando de 12,6 a 12,9 por 100 mil habitantes naquele período. Em Salvador observa-se tendência de redução, passando de 27,9 casos de aids por 100 mil hab., em 2009, para 24,4 casos por 100 mil hab. em 2019.^22^

A população do estudo constituiu-se de todas as pessoas que buscaram a PEP em Salvador, no período de 1^o^ de janeiro a 31 de dezembro de 2018. Os critérios de indicação para PEP foram: ter procurado o serviço em busca da PEP até 72 horas após a exposição; ter-se exposto e ser testado não reagente para o HIV; e ter a pessoa-fonte com testagem reagente para HIV ou com *status* sorológico desconhecido. Importante ressaltar que a testagem de HIV nas unidades de pronto atendimento (UPAs) era realizada aplicando-se um teste rápido de terceira geração, para detecção qualitativa de anticorpos contra o HIV. A aplicação do teste rápido tem a vantagem da execução simples, dispensa de estrutura laboratorial complexa, leitura de seus resultados a olho nu e conclusão em até 30 minutos. A janela de soroconversão dos ensaios de terceira geração é de aproximadamente 22 a 25 dias.^23^

De acordo com o protocolo de atendimento em PEP no município de Salvador, em 2018, quando o indivíduo era exposto ao vírus, ele se dirigia a uma das UPAs da cidade para avaliação inicial sobre tempo e tipo da exposição; em seguida, eram realizados os testes rápidos para HIV, sífilis, hepatite B e C, que também seriam ofertados à pessoa-fonte se estivesse presente. Concluída essa avaliação inicial, eram definidas as condutas quanto à prescrição da PEP para o HIV e/ou outras ISTs. Em seguida, eram dispensados os ARVs para 28 dias de uso, e o usuário era orientado e encaminhado para acompanhamento no Serviço Médico da Atenção Especializada (SEMAE), localizado no Distrito Sanitário Liberdade.

Para o acompanhamento e continuidade do tratamento, recomendava-se ao usuário que recebeu a prescrição da PEP comparecer a três consultas no SEMAE, agendadas para duas semanas, 30 e 90 dias após a data da exposição. Na primeira consulta, o usuário era avaliado quanto à toxicidade dos ARVs, com o objetivo de identificar possíveis efeitos adversos; e eram-lhe prestadas orientações no sentido de reforçar sua adesão à profilaxia. Nas consultas de 30 e 90 dias pós-exposição, eram realizadas novas testagens para HIV e reforçadas as orientações sobre medidas de prevenção à infecção, como o uso de preservativos em todas as relações sexuais e o não compartilhamento de seringas e agulhas (nos casos de uso de drogas injetáveis), além da contraindicação de doação de sangue, órgãos, tecidos ou esperma, e a reafirmação da importância de evitar a gravidez durante o tratamento.^8^ As consultas de seguimento no SEMAE eram realizadas por uma equipe multiprofissional, composta de assistente social, enfermeiro, farmacêutico, infectologista, psicólogo e técnico de enfermagem.

O estudo foi conduzido com base em dados secundários. O acesso e construção do banco de dados anonimizado desenvolveu-se em duas etapas.

Na primeira etapa, entre 2 de dezembro de 2019 e 31 de janeiro de 2020, construiu-se o banco de dados dos usuários que buscaram a PEP nas cinco UPAs com protocolo de PEP implantado no município de Salvador, no ano de 2018 (*i.e.*, Barris, Marback, Hélio Machado, Albergaria ou Valéria). As informações dos usuários estavam organizadas em Planilhas de Monitoramento da PEP e foram fornecidas pelo Setor de Acompanhamento das ISTs/Aids e Hepatites Virais, da Secretaria Municipal de Saúde de Salvador.

As planilhas eram preenchidas pelas enfermeiras dos núcleos de epidemiologia das UPAs, de acordo com as informações contidas nos prontuários de atendimento. Entre as informações registradas na planilha, destacam-se: nome do paciente, idade, sexo, data do atendimento/avaliação inicial de PEP, data provável da exposição ao HIV, tempo de exposição (período, em horas, da provável exposição até a busca pelo atendimento na UPA), tipo de exposição, testes rápidos realizados no atendimento e seus respectivos resultados, e prescrição da PEP. Não havia registro da identidade de gênero dos usuários. O protocolo padronizado de preenchimento das planilhas foi confeccionado pela Vigilância Epidemiológica da Secretaria Municipal de Saúde. O banco de dados desta etapa foi criado sobre a compilação de todas as informações catalogadas nas cinco UPAs, a partir da planilha. Foi compilado um total de 60 planilhas, referentes aos 12 meses do ano de 2018 (1^o^ de janeiro a 31 de dezembro de 2018), preenchidas nas cinco UPAs citadas previamente. O banco foi anonimizado, utilizando-se códigos identificadores sequenciais. Para a fase de pré-processamento da base de dados, foram utilizados os programas SPSSwin 22.0 e Excel.

A segunda etapa de construção do banco de dados consistiu de sua coleta nos prontuários do SEMAE, serviço onde ocorreram as consultas de acompanhamento. Foram coletados os registros da primeira, da segunda e da terceira consultas de acompanhamento da PEP. Nesta etapa, os usuários foram identificados nominalmente, para localização dos prontuários, e, em seguida, codificados com o código identificador atribuído na primeira etapa. Foi utilizado um instrumento de coleta, visando à padronização no registro dos dados. Foram coletados dados sociodemográficos, tipo de exposição ao HIV, nome da UPA onde se realizou o primeiro atendimento, e dados da primeira, da segunda e da terceira consultas. Esta coleta de dados considerou o período de 1^o^ de junho de 2020 a 23 de junho de 2020.

As variáveis analisadas foram:

a) prescrição da PEP (sim; não);

b) cumprimento das visitas de acompanhamento da PEP no SEMAE (sim; não);

c) sexo (masculino; feminino);

d) idade (em anos: menos de 18; 18 a 23; 24 a 33; 34 ou mais);

e) tipo de exposição (relação sexual consentida; exposição acidental a material biológico; violência sexual);

f) tempo de exposição (até 72 horas; mais de 72 horas);

g) resultado do teste de HIV na triagem (não reagente; reagente);

h) resultado do teste de sífilis na triagem (não reagente; reagente);

i) resultado do teste de hepatite B na triagem (não reagente; reagente);

j) resultado do teste de hepatite C na triagem (não reagente; reagente);

k) elegibilidade para PEP (elegível; não elegível);

l) motivos da não prescrição da PEP entre pessoas elegíveis (recusa; evasão; médico não prescreveu; falta do antirretroviral na UPA; sem informação);

m) efeitos adversos à PEP (náusea; diarreia; dor abdominal; cefaleia; vômito; icterícia; boca seca); e

n) manutenção da PEP (sim; suspensão após testagem da pessoa-fonte não reagente para o de HIV).

Realizou-se análise descritiva das variáveis de interesse, sendo obtidas frequências absolutas e percentuais. Para as análises estatísticas, utilizou-se o aplicativo Stata versão 12.0 (StataCorp LP, College Station, USA).

O acesso às planilhas e aos prontuários do SEMAE foi condicionado à assinatura do Termo de Anuência nº 79/2019, fornecido pela Coordenadoria de Gestão de Pessoas da Saúde, da Secretaria Municipal de Saúde de Salvador, autorizando o uso das informações para esta pesquisa. O projeto da pesquisa foi aprovado pelo Comitê de Ética em Pesquisa do Instituto de Saúde Coletiva da Universidade Federal da Bahia: Certificado de Apresentação para Apreciação Ética n^o^ 25525719.6.0000.5030; Parecer n^o^ 3.746.970. A confidencialidade dos dados primários, aprovada em 5 de dezembro de 2019, foi garantida durante a coleta; e o banco de dados resultante, anonimizado, foi trabalhado em computador privado, sem acesso à internet, de uso restrito e protegido por senha, de maneira a proteger as informações.

## RESULTADOS

Dos 1.550 atendimentos de PEP registrados em 2018, foram excluídos 25 por se referirem ao registro de pessoas-fonte de indivíduos que sofreram acidente com material biológico e, portanto, não necessitavam de PEP. Das 1.525 pessoas que buscaram a PEP em UPAs no município de Salvador, 1.056 (69,2%) eram do sexo masculino e 469 (30,8%) do sexo feminino. A mediana de idade dessas pessoas foi de 28 anos (intervalo interquartil: 22 a 35), com predominância dos atendimentos na faixa etária dos 24 aos 33 anos (39,4%). Entre crianças e adolescentes < 18 anos, verificou-se um total de 81 atendimentos (5,3%) (Tabela 1), dos quais 37 (45,7%) foram devidos a violência sexual, 24 (29,6%) por relação sexual consentida e 20 (24,7%) por acidente com agulhas.

**Tabela 1:** Características demográficas e de exposição à infeção pelo vírus da imunodeficiência humana (HIV) e procedimentos realizados durante a avaliação da profilaxia pós-exposição de risco à infeção, Salvador, Bahia, 2018

Características	N	%
Sexo		
Masculino	1.056	69,2
Feminino	469	30,8
Idade (anos)		
< 18	81	5,3
18-23	398	26,1
24-33	601	39,4
≥ 34	440	28,9
Sem informação	5	0,3
Tipo de exposição ao HIV		
Relação sexual consentida	1.164	76,3
Exposição acidental a material biológico	270	17,7
Violência sexual	86	5,6
Sem informação	5	0,3
Tempo de exposição (horas)		
≤ 72	1.381	90,6
> 72	93	6,1
Sem informação	51	3,3
HIV		
Não reagente	1.403	92,0
Reagente	25	1,6
Não realizado	97	6,4
Sífilis		
Não reagente	1.265	83,0
Reagente	156	10,2
Não realizado	104	6,8
Hepatite B		
Não reagente	1.407	92,3
Reagente	2	0,1
Não realizado	116	7,6
Hepatite C		
Não reagente	1.408	92,3
Reagente	2	0,1
Não realizado	115	7,5
Indicação para profilaxia pós-exposição (PEP) ao HIV		
Sim	1.273	83,5
Não	252	16,5
Prescrição da PEP		
Sim	1.166	91,6
Não	107	8,4
Motivos de não prescrição da PEP entre pessoas com indicação		
Recusa	42	39,2
Evasão	37	34,6
Médico não prescreveu	22	20,6
Falta do antirretroviral na unidade de pronto atendimento	3	2,8
Sem informação	3	2,8

Em relação ao tipo de exposição que motivou a busca pela PEP, 1.164 (76,3%) pessoas relataram contato sexual desprotegido; 270 (17,7%), exposição acidental a material biológico; e 86 (5,6%), violência sexual. Entre os homens, a exposição que motivou a busca por PEP foi o contato sexual desprotegido (938 = 88,8%), acidente com exposição a material biológico (101 = 9,6%), violência sexual (12 = 1,1%) e não registro do motivo da busca (5 = 0,5%); e entre as mulheres, a exposição e motivo da busca foi o contato sexual desprotegido (226 = 48,2%), acidente com exposição a material biológico (169 = 36%) e violência sexual (74 = 15,8%) (Tabela 1).

O tempo decorrido do momento da exposição até a chegada à UPA foi menor ou igual a 72 horas para 1.381 (90,6%); 93 (6,1%) procuraram a PEP passado esse período. Entre todos e todas, 1.403 (92,0%) tiveram teste não reagente para HIV durante o primeiro atendimento; o resultado do teste foi reagente em apenas 25 (1,6%), logo encaminhadas para tratamento no SEMAE. Finalmente, 156 (10,2%) tiveram teste reagente para sífilis, 2 (0,1%) para hepatite B e 2 (0,1%) para hepatite C (Tabela 1).

Do total de pessoas que buscaram PEP, 1.273 (83,5%) apresentaram os critérios de elegibilidade para início imediato, enquanto 252 (16,5%) não os apresentaram. Entre os motivos de não elegibilidade, a maioria buscou o serviço após 72 horas da exposição (93 = 36,9%). Cumpre observar que 25 receberam resultado reagente para HIV no primeiro atendimento (9,9%), 84 tiveram a pessoa-fonte testada não reagente para o vírus (33,3%) e 50 não apresentavam registro do motivo de não elegibilidade no prontuário (19,9%).

Considerando-se as pessoas que tinham indicação para PEP, 1.166 (91,6%) receberam a prescrição dos ARVs e foram orientadas a seguir com o acompanhamento previsto no protocolo. Entretanto, 107 (8,4%) delas não receberam a prescrição, apesar de apresentarem os critérios de indicação para PEP, e os motivos da não prescrição foram: 42 (39,2%) recusaram a profilaxia; 37 (34,6%) evadiram da unidade; 22 (20,6%) não receberam do médico a prescrição de antirretroviral; e 3 (2,8%) ficaram sem a prescrição, por falta de medicamento na UPA (Tabela 1).

Das pessoas que receberam a prescrição da PEP (1.166), 226 (19,4%) compareceram à primeira consulta, 115 (9,9%) à segunda consulta e 33 (2,8%) à terceira consulta para conclusão do protocolo (Figura 1). Todos os usuários que compareceram às consultas de acompanhamento realizaram a testagem para HIV nessas ocasiões, com resultados não reagentes. A maioria que compareceu à primeira consulta de acompanhamento no SEMAE era do sexo masculino (64,6%), idade de 34 anos ou mais (37,2%), e expusera-se ao risco de infecção por relação sexual consentida (70,4%). Nesta primeira consulta, as reações adversas aos ARVs mais frequentes foram náusea (32,7%), diarreia (19,9%), dor abdominal (7,1%), cefaleia (6,2%), vômito (3,5%), icterícia (3,5%) e boca seca (2,2%). A maioria teve adesão ao esquema antirretroviral (96,9%) e apenas 3,1% tiveram indicação de suspensão da PEP devido à testagem da pessoa-fonte ter resultado não reagente para o HIV (Tabela 2).

**Tabela 2:** Características demográficas e de exposição à infeção pelo vírus da imunodeficiência humana (HIV) e relativas à primeira consulta de acompanhamento no Serviço Médico da Atenção Especializada, e frequência de comparecimento às consultas, Salvador, Bahia, 2018

Características	N	%
Sexo		
Masculino	146	64,6
Feminino	80	35,4
Idade (anos)		
< 18 18-23	13 53	5,8 23,5
24-33	76	33,6
≥ 34	84	37,2
Tipo de exposição		
Relação sexual consentida	159	70,4
Exposição acidental a material biológico	54	23,9
Violência sexual	13	5,8
Efeitos adversos		
Náusea	74	32,7
Diarreia	45	19,9
Dor abdominal	16	7,1
Cefaleia	14	6,2
Vômito	8	3,5
Icterícia	8	3,5
Boca seca	5	2,2
Manutenção da profilaxia pós-exposição (PEP) ao HIV		
Sim	219	96,9
Suspensão após testagem da pessoa-fonte não reagente para o HIV	7	3,1
1ª consulta de acompanhamento	226	19,4
2ª consulta de acompanhamento	115	9,9
3ª consulta de acompanhamento	33	2,8

**Figura 1:** Prescrição da profilaxia pós-exposição de risco à infeção pelo vírus da imunodeficiência humana e retorno às consultas de acompanhamento, Salvador, Bahia, 2018

a) PEP: Profilaxia pós-exposição ao HIV.

## DISCUSSÃO

A PEP é uma estratégia de prevenção segura e cumpre um papel ainda mais amplo, na testagem de HIV e diagnóstico de outras ISTs. Neste estudo, ao se analisar a prescrição de PEP e o retorno às consultas de acompanhamento no município de Salvador, em 2018, os resultados descreveram a busca pela PEP nas UPAs, principalmente por pessoas mais jovens (24 a 33 anos), do sexo masculino, expostas à infecção em relação sexual consentida. Entretanto, o acompanhamento subsequente no SEMAE registrou um perfil de pessoas mais velhas (34 anos ou mais). Além disso, o percentual de pessoas que cumpriram todo o protocolo de PEP à época, qual seja, a realização das três consultas de acompanhamento, reduziu-se ao longo do tempo, de 19,4% para 2,8% do total de pessoas que iniciaram a profilaxia.

Os achados desta pesquisa corroboram os de outros estudos, ao se demonstrar que os métodos de prevenção baseados em ARVs são mais procurados por pessoas do sexo masculino, jovens, com média de idade semelhante.^10^ ^),(^ ^24^ Estudo de coorte realizado em Tel Aviv, Israel, entre janeiro de 2013 e junho de 2014, identificou 75,9% das pessoas que buscaram a PEP como homens que fazem sexo com homens adultos e com uma idade média de 32,4 anos.^24^ Neste estudo de Salvador, a exposição sexual também foi o principal motivo da procura pela PEP, corroborando outro estudo, este realizado em clínica comunitária na cidade de Los Angeles, Estados Unidos, entre março de 2010 e julho de 2014.^25^

Apesar de a PEP ser uma estratégia de prevenção já consolidada, alguns usuários do sistema de saúde recusaram-na ou se evadiram da unidade, enquanto, noutros casos, o profissional médico não prescreveu ARVs. Estes achados apontam para falhas no acolhimento pelos serviços de saúde; o mesmo que apontou outro estudo, realizado com 4.188 mulheres trabalhadoras do sexo, recrutadas por amostragem baseada no participante, em 12 cidades brasileiras, no ano de 2016. Nesse estudo, 1.199 mulheres relataram ter sofrido violência sexual e apenas 7,5% delas procuraram assistência médica e utilizaram PEP; e 19%, ainda que tenham procurado assistência médica, não utilizaram a PEP.^26^

A construção de programas de treinamento para os profissionais de saúde das UPAs e de outros serviços que ofertam PEP no SUS é importante, de modo a que eles considerem essa tecnologia, tanto como uma urgência médica como um processo, e recomendem uma atitude profissional de acolhimento, sem julgamentos morais.^27^ ^),(^ ^28^

No que concerne às consultas de acompanhamento, os achados da presente pesquisa revelam uma queda entre a prescrição da PEP e as consultas de acompanhamento no serviço especializado de referência. A falha no acompanhamento pode implicar dificuldades para o acompanhamento, e adesão à PEP^2^ ^)-(^ ^4^ ^)^ durante os 28 dias, e afinal, o sucesso da estratégia.^8^ Importante ressaltar que a UPA e o SEMAE não realizavam busca ativa dos usuários ausentes às consultas de acompanhamento, uma ação que poderia contribuir para a efetividade da PEP.

Embora não fosse possível identificar as razões das perdas de acompanhamento, há a hipótese de que a fragmentação do cuidado em PEP no município de Salvador, isto é, a oferta inicial realizada em uma unidade e o seguimento em outra - muitas vezes separadas por quilômetros de distância - tivesse papel importante na não vinculação do paciente ao serviço especializado de seguimento. Estudo realizado na cidade de Boston, Estados Unidos, no ano de 2014, verificou que pessoas atendidas em unidade de emergência e que recebiam 3 a 6 dias de esquema antirretroviral para PEP, posteriormente encaminhadas para acompanhamento em clínica especializada, tiveram baixa adesão a este serviço.^29^ Diversos fatores podem explicar essa dificuldade, como as características dos serviços de saúde, a qualidade do cuidado, a relação do profissional de saúde com o usuário, além da acessibilidade do serviço.

Este estudo possui algumas limitações. Ao se basear em dados secundários, extraídos de planilhas e prontuários preenchidos por profissionais de serviços de saúde que atendem as demandas de uma unidade de emergência, a qualidade das informações está condicionada, por um lado, à disposição e julgamento dos profissionais, ao inquirirem e registrarem informações importantes e, por outro lado, à disposição dos indivíduos para relatar informações íntimas. Além disso, foi pequeno o número de variáveis disponível, visto que os dados provieram de registros dos serviços e não de pesquisa delineada previamente, para coleta desses dados. Desse modo, o banco de dados construído para o estudo pode não contar com variáveis importantes de serem analisadas, como a identidade de gênero das pessoas e aspectos de acessibilidade aos serviços especializados. Contudo, os resultados encontrados apontam para a necessidade de aumento da divulgação de informações sobre a PEP, visto que o município oferece o tratamento de forma gratuita no SUS, em UPAs abertas 24 horas por dia.

Em conclusão, o presente estudo indica a necessidade de reavaliação do processo de implementação do protocolo clínico e diretrizes terapêuticas na capital soteropolitana, mediante qualificação dos profissionais encarregados do atendimento em PEP, divulgação dos locais onde as pessoas têm acesso à profilaxia e designação de uma equipe para o monitoramento daqueles em uso da PEP, via ligações telefônicas e/ou aplicativos, de modo a expandir e qualificar o acesso às tecnologias de tratamento baseadas em ARVs. São, justamente, ações que podem contribuir para a consolidação dos princípios e o fortalecimento do SUS, o enfrentamento e a superação de estigmas sociais, reafirmando a luta política pelo direito à saúde em Salvador e no Brasil.^30^
